# Antenatal Corticosteroids: Short-Term Gains, Long-Term Questions—A Meta-Analysis

**DOI:** 10.3390/ph19071098

**Published:** 2026-07-17

**Authors:** Marharyta Sobczak, Barbara Wencka, Rafał Pawliczak

**Affiliations:** 1 Department of Immunopathology, Division of Biomedical Science, Faculty of Medicine, Medical University of Lodz, 90-752 Lodz, Poland; marharyta.sobczak@umed.lodz.pl; 2 Department of Perinatology, Obstetrics and Gynecology, Polish Mother’s Health Center Institute, 93-338 Lodz, Poland; basia.wencka@gmail.com

**Keywords:** antenatal corticosteroids, disorders, long-term effect, neurological effects, premature birth

## Abstract

**Background/Objectives**: Antenatal corticosteroids (ACSs) form the basis of prenatal care for women at risk of preterm birth, primarily due to their proven benefits in reducing neonatal mortality and short-term morbidity. However, there are growing concerns about the potential long-term consequences of fetal exposure to ACSs. The aim of the meta-analysis was to assess the relationship between exposure to corticosteroids administered during the prenatal period and long-term effects. **Methods**: PubMed, Web of Science, and the Cochrane Central Register of Controlled Trials were searched for studies published up to 28 January 2026. Randomized controlled trials and observational studies reporting long-term outcomes after exposure to ACSs were included. A random effects model was used to calculate effect sizes. **Results**: Among the neurological disorders analyzed, our meta-analysis showed an increased risk of 17% of suspected neurocognitive disorder (RR = 1.17; 95% CI [1.13; 1.21]; *p* < 0.0001, I^2^ = 10%), 18% of hearing impairment (RR = 1.18; 95% CI [1.13; 1.24]; *p* < 0.0001, I^2^ = 41%), 3% of visual impairment (RR = 1.03; 95% CI [1.01; 1.05]; *p* = 0.0012, I^2^ = 0%), 25% of autism spectrum disorders (RR = 1.25; 95% CI [1.09; 1.42]; *p* = 0.001, I^2^ = 43%) and 25% of any mental or behavioral disorder (RR = 1.25; 95% CI [1.1; 1.43]; *p* = 0.0008, I^2^ = 77%). The results of other neurological disorders, as well as other analyzed outcomes, were not statistically significant (*p* > 0.05). **Conclusions**: Although this meta-analysis suggests an increased risk of certain long-term neurological outcomes following prenatal exposure to corticosteroids, these findings should be interpreted with caution in light of the well-documented short-term benefits for newborns and the need for a careful assessment of the benefit–risk ratio and further long-term observational studies.

## 1. Introduction

As defined by the WHO, preterm birth is the birth of a live baby before 37 weeks of gestation, classified as extremely preterm (<28 weeks), very preterm (28 to <32 weeks), moderate (32 to <34 weeks), and late preterm (34 to <37 weeks). Although a baby born after 37 weeks of pregnancy is not considered premature, it is recommended to continue the pregnancy until 39 weeks if there are no contraindications. Every year, around 15 million babies are born prematurely. Over 84% of preterm infants are born between 32 and 36 weeks of gestation, while approximately 5% are classified as extremely preterm, having been born before 28 weeks of gestation [1]. Premature birth represents a major cause of prenatal hospitalization and the leading cause of mortality, and accounts for approximately 70% of neonatal deaths and 36% of infant deaths. Furthermore, prematurity accounts for an estimated 25–50% of cases of long-term neurological disorders in children [2].

The administration of corticosteroids before expected preterm birth is one of the key prenatal interventions that significantly improve the prognosis for newborns [3]. Prematurity is still a controversial topic, including the choice of corticosteroid, dosage, timing of administration, and use of repeated doses. Betamethasone and dexamethasone are the most commonly prescribed corticosteroids for preventing morbidity and mortality associated with preterm birth. They are recommended by international guidelines in cases of imminent preterm birth, and the recommended total dose of dexamethasone or betamethasone is 24 mg intramuscularly. The issue of repeated dosing remains controversial when preterm birth does not occur as anticipated after corticosteroid administration. Bedside tests used to predict preterm birth have high negative but low positive predictive values, resulting in widespread use of prenatal corticosteroids. However, retrospective studies indicate that only about 33% of treated women give birth before 34^+0^ weeks of pregnancy [4]. Of course, it should be emphasized that antenatal corticosteroid (ACS) therapy is best known for inducing accelerated functional maturation of the lungs of prematurely born infants, which leads to a reduction in the risk of mortality and morbidity in newborns, including respiratory distress syndrome, intraventricular hemorrhage, and necrotizing enterocolitis. However, there are also concerns about the potential risk of adverse health effects or exposing the fetus to unnecessarily high doses of corticosteroids. Short-term effects of ACS exposure include an increased incidence of neonatal hypoglycemia, as well as an elevated risk of infections in infancy, particularly during the first six months of life. Data on the long-term consequences of ACS exposure are limited, but potential neurological, endocrine, and cardiovascular effects have been detected [5]. Therefore, we decided to conduct a meta-analysis of studies to investigate the effects of long-term exposure to ACSs.

## 2. Results

### 2.1. Search Results

**Figure 1:**
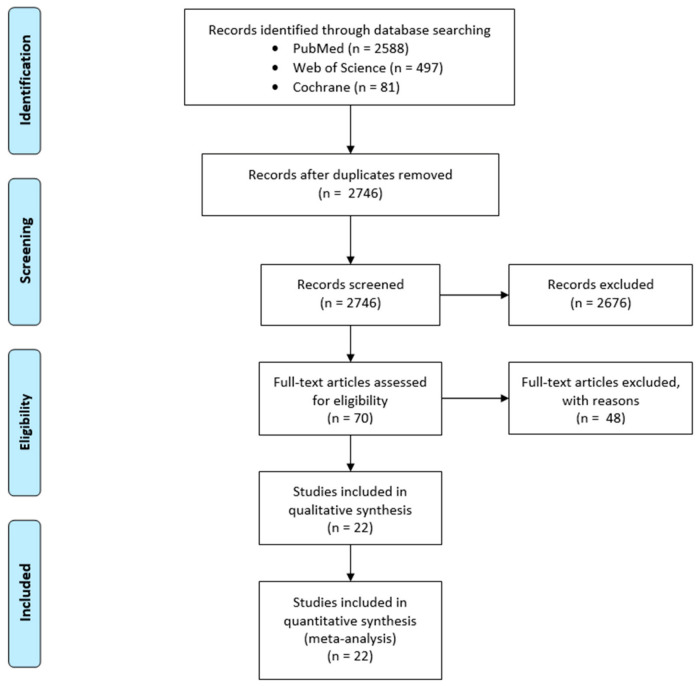
Study selection for meta-analysis.

The literature search yielded 2746 articles after removal of duplicates (Figure 1). In the first screening, we excluded 2676 articles, such as meta-analysis, systematic reviews, literature reviews, and editorial letters, as well as in vitro studies, studies on animals and case reports. After full-text screening, 22 articles were qualified for meta-analysis. As shown in Table 1, RCTs and cohort studies from various countries were included in the analysis.

### 2.2. Quality Assessment

All randomized controlled trials were judged to be at low risk of bias (Figure S1), while all cohort studies were assessed as being of high methodological quality (Table S1).

### 2.3. Long-Term Neurological Effect Following Antenatal Corticosteroid Exposure

**Figure 2:**
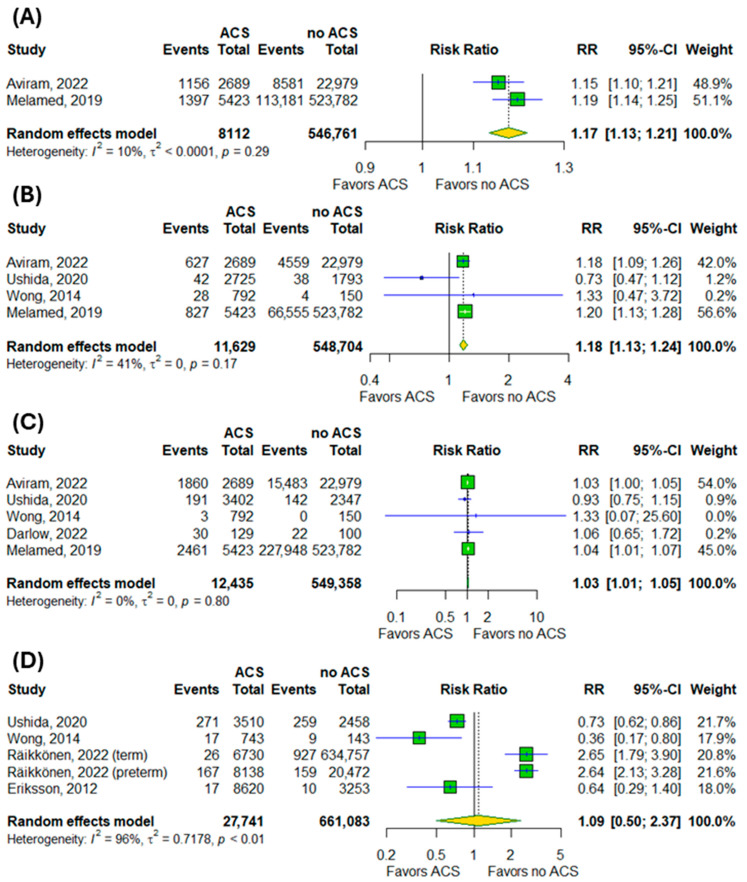
Long-term effect of ACSs on neurological outcomes: (**A**) suspected neurocognitive disorder, (**B**) hearing and (**C**) visual impairment, (**D**) and cerebral palsy [6,9,10,17,21,23,27].

**Figure 3:**
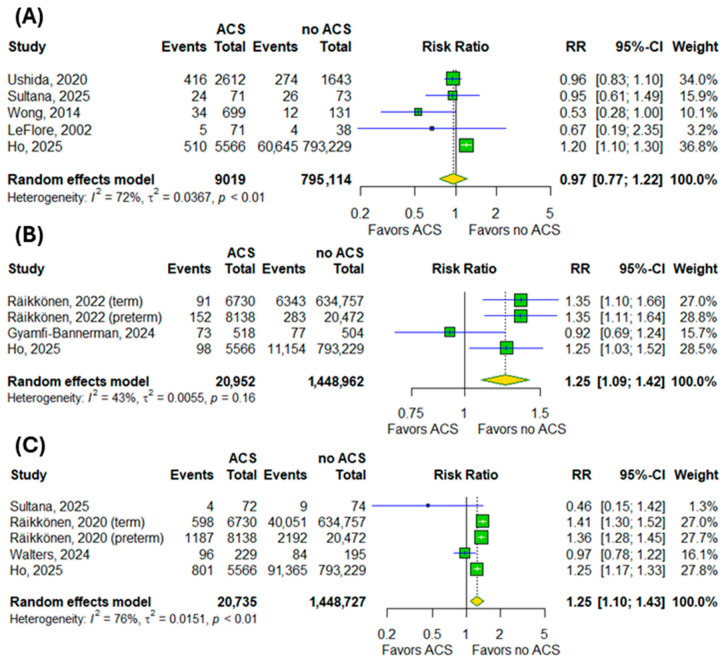
Long-term effect of ACSs on neurological outcomes: (**A**) neurodevelopmental impairment, (**B**) autism spectrum disorders and (**C**) any mental or behavioral disorder [11,12,14,20,21,22,23,26,27].

We analyzed a total of 13 articles to examine the long-term neurological effects of ACS use, as shown in Figure 2A–D and Figure 3A–C. Among the neurological disorders analyzed, our meta-analysis showed an increased risk of 17% of suspected neurocognitive disorder (RR = 1.17; 95% CI [1.13; 1.21]; *p* < 0.0001, I^2^ = 10%), 18% of hearing impairment (RR = 1.18; 95% CI [1.13; 1.24]; *p* < 0.0001, I^2^ = 41%), 3% of visual impairment (RR = 1.03; 95% CI [1.01; 1.05]; *p* = 0.0012, I^2^ = 0%), 25% of autism spectrum disorders (RR = 1.25; 95% CI [1.09; 1.42]; *p* = 0.001, I^2^ = 43%) and 25% of any mental or behavioral disorder (RR = 1.25; 95% CI [1.1; 1.43]; *p* = 0.0008, I^2^ = 77%). The risk of other neurological disorders, such as cerebral palsy (RR = 1.09; 95% CI [0.5; 2.37]; *p* = 0.8335, I^2^ = 97%) and neurodevelopmental impairment (RR = 0.97; 95% CI [0.77; 1.22]; *p* = 0.7757, I^2^ = 72%), was analyzed.

### 2.4. Long-Term Anthropometric Effect Following Antenatal Corticosteroid Exposure

**Figure 4:**
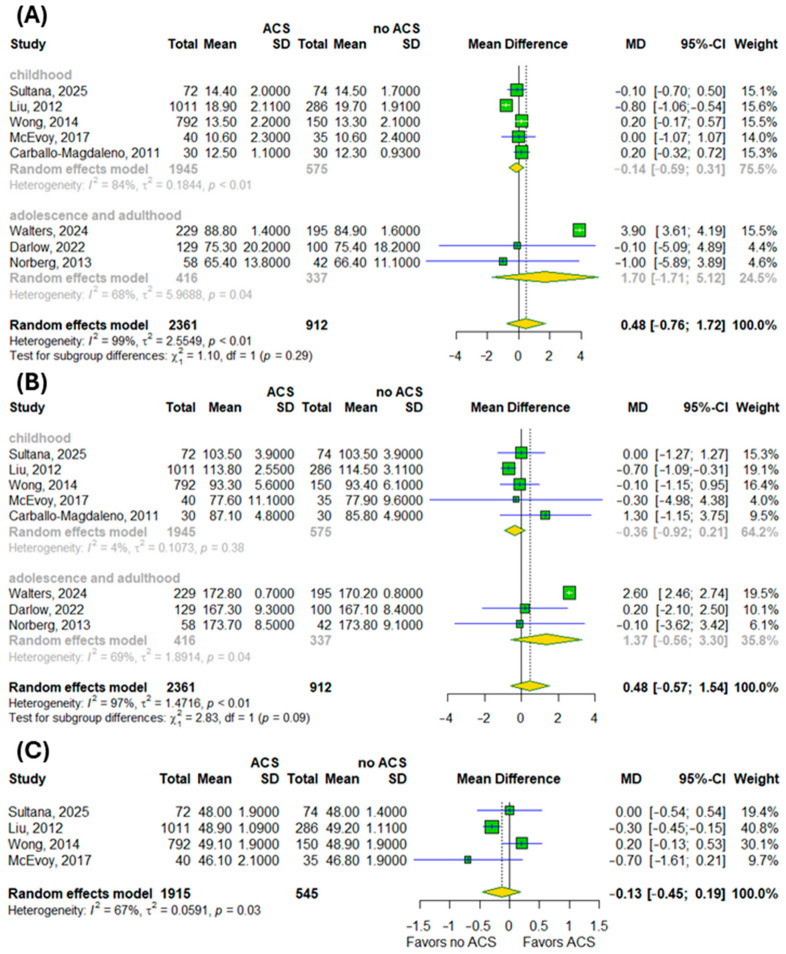
Long-term effect of ACSs on anthropometric outcomes: (**A**) weight, (**B**) height and (**C**) head circumference [7,9,15,16,19,22,25,27].

Further analyses were conducted to examine the effect of ACSs on anthropometric parameters, such as weight (MD = 0.48; 95% CI [−0.76; 1.72]; *p* = 0.4502, I^2^ = 99%), height (MD = 0.48; 95% CI [−0.57; 1.54]; *p* = 0.3684, I^2^ = 97%) and head circumference (MD = −0.13; 95% CI [−0.45; 0.19]; *p* = 0.4229, I^2^ = 67%) (Figure 4A–C). Furthermore, in the case of weight and height, an analysis of subgroups by age range was carried out, which also did not reveal any significant differences (*p* > 0.05).

### 2.5. Long-Term Cardiovascular Effect Following Antenatal Corticosteroid Exposure

**Figure 5:**
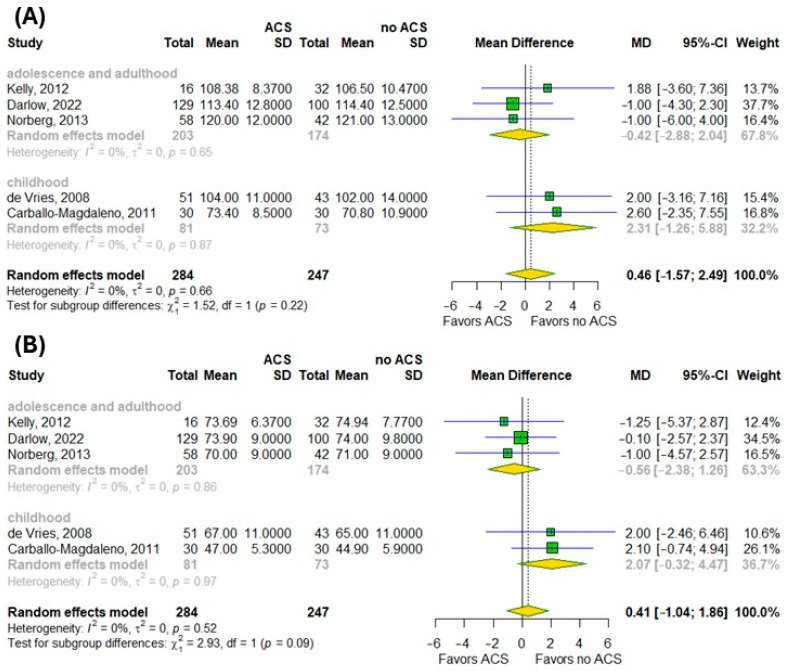
Long-term effect of ACSs on cardiovascular outcomes: (**A**) systolic blood pressure and (**B**) diastolic blood pressure [7,9,13,19,24].

In the case of effects on the cardiovascular system, systolic (MD = 0.46; 95% CI [−1.57; 2.49]; *p* = 0.656, I^2^ = 0%) and diastolic (MD = 0.41; 95% CI [−1.04; 1.87]; *p* = 0.5835, I^2^ = 0%) blood pressure were analyzed, including subgroup analyses (Figure 5A,B).

### 2.6. Long-Term Metabolic Effect Following Antenatal Corticosteroid Exposure

**Figure 6:**
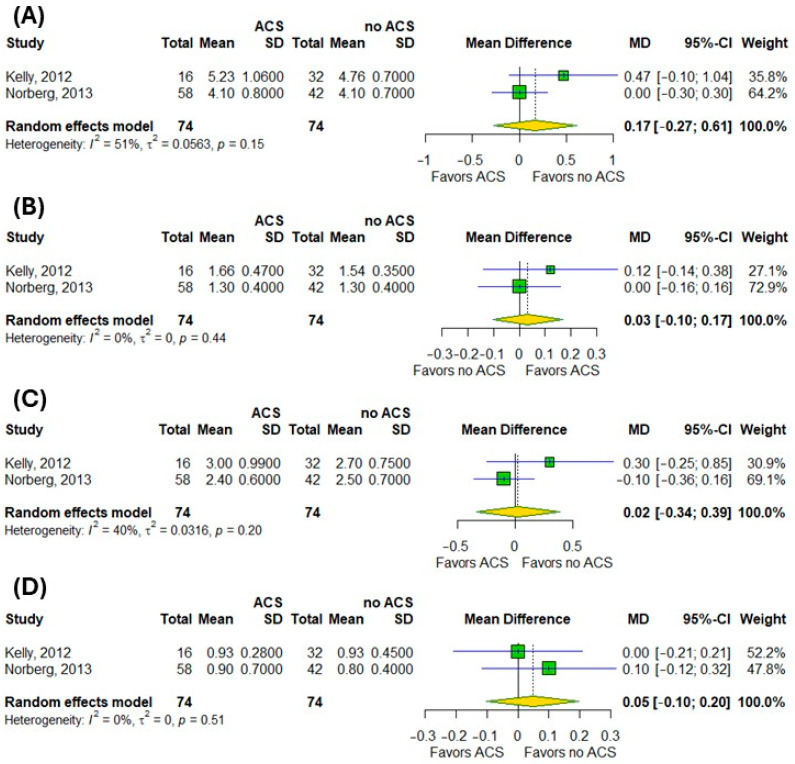
Long-term effect of ACSs on metabolic outcomes: (**A**) cholesterol, (**B**) HDL, (**C**) LDL and (**D**) triglycerides [13,19].

As regards the metabolic effect after exposure to ACSs, we analyzed the lipid profile. The analysis was based on data from two studies and did not reveal any significant differences in cholesterol (MD = 0.17; 95% CI [−0.27; 0.61]; *p* = 0.4554, I^2^ = 51%), HDL (MD = 0.03; 95% CI [−0.1; 0.16]; *p* = 0.6378, I^2^ = 0%), LDL (MD = 0.02; 95% CI [−0.34; 0.39]; *p* = 0.8976, I^2^ = 40%) and triglyceride (MD = 0.05; 95% CI [−0.1; 0.2]; *p* = 0.5323, I^2^ = 0%) levels (Figure 6A–D).

### 2.7. Long-Term Respiratory Effect Following Antenatal Corticosteroid Exposure

**Figure 7:**
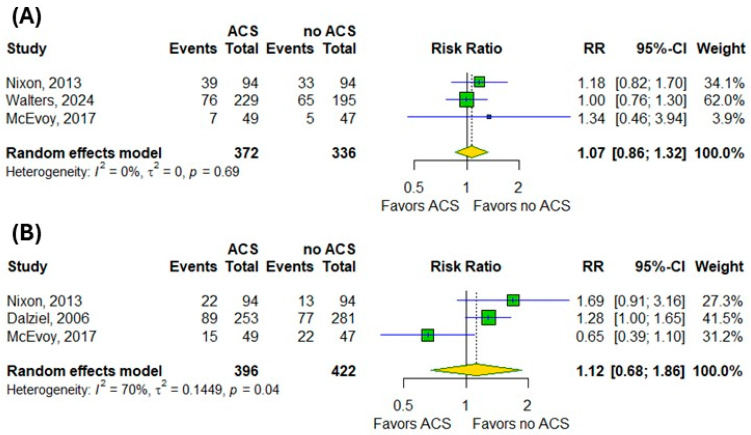
Long-term effect of ACSs on respiratory outcomes: (**A**) asthma and (**B**) wheezing [8,16,18,25].

Similar results were observed for respiratory outcomes, such as asthma (RR = 1.07; 95% CI [0.86; 1.32]; *p* = 0.5451, I^2^ = 0%) and wheezing (RR = 1.12; 95% CI [0.68; 1.86]; *p* = 0.6573, I^2^ = 70%), based on data from three studies (Figure 7A,B).

### 2.8. Publication Bias

Publication bias was assessed using funnel plots (Figure S2) and Egger’s and Peters’ regression tests. The results indicated no evidence of publication bias for the association between ACS exposure and any of the assessed outcomes: hearing impairment (*p* = 0.73), visual impairment (*p* = 0.89), cerebral palsy (*p* = 0.17), neurodevelopmental impairment (*p* = 0.46), autism spectrum disorders (*p* = 0.41), any mental or behavioral disorder (*p* = 0.72), weight (*p* = 0.81), height (*p* = 0.18), head circumference (*p* = 0.68), systolic blood pressure (*p* = 0.16), diastolic blood pressure (*p* = 0.87), asthma (*p* = 0.16), and wheezing (*p* = 0.53). Tests for other outcomes could not be performed due to the limited number of included studies.

## 3. Discussion

In our meta-analysis, we observed statistically significant associations between ACS exposure and increased risk of neurological disorders, such as suspected neurocognitive disorder, hearing and visual impairments, autism spectrum disorders and any mental or behavioral disorder. In contrast, we did not show significant results for other neurological outcomes (cerebral palsy and neurodevelopmental impairment), as well as other analyzed outcomes, such as anthropometric, cardiovascular, metabolic and respiratory. However, given the limited number of studies and the imprecision of the estimates reflected by wide confidence intervals, these findings should be interpreted with caution.

In 1969, a study in sheep demonstrated partial lung expansion in lambs treated with corticosteroids prenatally, suggesting accelerated surfactant production and providing early mechanistic evidence of lung maturation [28]. Premature birth is a major risk factor for brain injury and abnormalities in nervous system development. Although ACS therapy is associated with a significant reduction in neonatal mortality and the incidence of retinopathy of prematurity, respiratory distress syndrome, and severe neurological complications in premature infants, its long-term protective role remains controversial. Furthermore, physiological concentrations of corticosteroids are crucial for normal fetal brain development, including the regulation of neurogenesis. At the same time, cortisol concentrations in the fetus must remain several times lower than in the mother for these processes to proceed normally, as excessive exposure to corticosteroids can lead to neurotoxicity. ACSs are able to cross the placenta and may directly affect fetal brain development, particularly when exposure occurs in later stages of pregnancy [29]. Evidence from a scoping review suggests a potential association between ACS exposure and neurological outcomes in preterm and term infants [30]. Moreover, a systematic review of ACS therapy in animal models detected harmful, long-lasting neurocognitive effects. However, analyzed studies varied and there was a high risk of bias [31]. Prenatal exposure to dexamethasone has been shown to lead to changes in the expression of glucocorticoid receptor and calcyon genes in the prefrontal cortex of common marmoset monkeys. The prefrontal cortex is a brain region implicated in ADHD, while calcyon has been associated with ADHD-related neurodevelopmental processes [32]. A prospective observational study of children exposed in utero to single or multiple courses of betamethasone for threatened preterm labor found no alterations in hypothalamic–pituitary–adrenal axis activity, but suggested potential effects on autonomic nervous system activity. Interestingly, although IQ scores were within the normal range, exposed children had lower scores [33].

In women with chorioamnionitis, the use of ACSs was associated with reduced neonatal mortality and a lower incidence of respiratory distress syndrome, intraventricular hemorrhage and severe intraventricular hemorrhage in pooled analyses [34]. The meta-analysis by Peltoniemi et al. [35] showed that ACS administration—whether as a single course or as weekly or biweekly repeat doses—reduced the risk of respiratory distress syndrome; however, repeated dosing was associated with significantly reduced intrauterine growth in preterm infants. However, no abnormalities in neurological development or growth were observed. Another meta-analysis by Deshmukh et al. [36] demonstrated that exposure to ACSs reduced the need for respiratory support and postnatal resuscitation in infants born between 34 and 36^+6^ weeks of gestation. However, it was associated with an increased risk of hypoglycemia in newborns. However, a retrospective observational cohort study in Korea found that administering ACSs in late preterm infancy (after 34 weeks) did not increase the risk of hypoglycemia or affect neurological development [37]. Interestingly, the study by Guerini et al. [38] demonstrated that the timing of ACS administration had a significant impact on the risk of neurologic disabilities. An interval of more than 7 days between antenatal corticosteroid administration and delivery was associated with a lower rate of survival without moderate or severe neurodevelopmental impairment at 5.5 years of age.

The use of multiple courses of ACSs is associated with adverse growth effects in newborns, including lower birth weight, shorter body length, and reduced head circumference compared to placebo. Interestingly, all women received an initiation course of ACSs prior to randomization [39]. Similar observations were also noted in a study of twin pregnancies, in which the use of ACSs was associated with adverse changes in the development of infants and young children [40]. Although no statistically significant differences in anthropometric measurements were observed in our meta-analysis, a pattern suggesting age-dependent changes can be noted. In childhood, the pooled mean differences for the analyzed outcomes ranged from −0.13 to −0.36, indicating a tendency toward lower values. In contrast, in adolescence and adulthood, the mean differences were positive, reaching 1.7 for body weight and 1.37 for height.

This meta-analysis has several important limitations that should be considered when interpreting the results. First, the included studies were highly heterogeneous in terms of study design, population characteristics, exposure definitions (including corticosteroid type, dosing regimen, and number of treatment courses), and outcome assessments. Long-term outcomes were assessed at different ages using inconsistent measurement protocols and diagnostic criteria, which may have contributed to the variability in effect estimates. Second, the included studies reported both cumulative incidence rates and point prevalence rates. This lack of consistency in the definitions of outcomes may have contributed to the heterogeneity observed across studies and should be taken into account when interpreting the results. Third, the definition of “long-term outcomes” was inconsistent across studies. Follow-up periods varied considerably, limiting direct comparability. Fourth, the studies included in the analysis were heterogeneous in terms of gestational age at delivery. Some cohorts consisted exclusively of individuals born prematurely, while others included participants born at term or mixed populations. Since premature birth itself is associated with long-term changes, this heterogeneity may have influenced the observed associations and limits the ability to attribute the results solely to the exposure of interest. Fifth, high heterogeneity was observed in some of the analyses, indicating significant variability across studies and limiting the ability to interpret the pooled effect as a single, unified estimate. Therefore, these results should be interpreted with caution. Finally, most included studies were observational in nature, limiting causal inference, because residual confounding, including confounding by indication, cannot be excluded. Despite pooling data to increase statistical power, the findings should be interpreted as associations rather than evidence of causality. Although this meta-analysis provides a comprehensive summary of long-term outcomes following ACSs, the results should be interpreted with caution due to heterogeneity in gestational age, study design, follow-up duration, and outcome assessment, as well as the possibility of residual confounding factors and selective reporting.

## 4. Materials and Methods

### 4.1. Search Strategy

This meta-analysis was conducted according to the PRISMA 2020 Statement [41]. PubMed, Web of Science and the Cochrane Central Register of Controlled Trials databases were searched for studies published up to 28 January 2026. The following keywords were used in the search strategy: antenatal, prenatal, neonatal, steroid, glucocorticoid, corticosteroid, betamethasone, dexamethasone, budesonide, preterm, premature, and long-term effects.

### 4.2. Study Selection and Data Extraction

The inclusion criteria covered all articles that contained information on the long-term effects of exposure to ACSs compared to non-exposure. The meta-analysis included both studies reporting the cumulative risk of a given outcome during the observation period and studies presenting the point prevalence at a specific age. The articles had to contain the following outcomes:

Neurological outcomes:

- Suspected neurocognitive disorder—any physician service claim with a diagnosis code related to a suspected neurocognitive disorder;
- Hearing impairment—physician service claim for audiometry testing outside the routine provincial infant screening program for hearing deficits, or bilateral hearing loss, or the need for hearing aids;
- Visual impairment—any consultations or assessments from an ophthalmologist or optometrist, or blindness with no functional vision in at least one eye or bilateral amblyopia, or bilateral blindness, or moderate visual impairment (any of the following: visual acuity (logMAR) > 0.3, better eye; myopia > 2.0 dioptres, better eye; hypermetropia > 2.0 dioptres, better eye; astigmatism > 2.0 dioptres, better eye);
- Cerebral palsy—cerebral palsy or cerebral palsy requiring walking aids;
- Neurodevelopmental impairment—developmental quotient < 70 or <−2 SD according to the Kyoto Scale of Psychological Development or Griffiths Mental Development Scales, scored by General Quotient or the Mental Scale of the Bayley Scales of Infant Development-II, scored by Mental Development Index (MDI); or Bayley Scales of Infant Development scored by MDI; or according to ICD-9-CM or ICD-10-CM; or abnormal results in ‘Problem Solving’ according to Ages & Stages Questionnaires, Third Edition (ASQ-3) scores;
- Autism spectrum disorders—autism, or autism spectrum disorders, or pervasive developmental disorders according to ICD-9-CM (299) and ICD-10-CM/PCS (F84), or Social Responsiveness Scale t score > 65;
- Any mental or behavioral disorder—any mental or behavioral disorder, or any childhood mental disorders, any behavioral or emotional problems according to the Strengths and Difficulties Questionnaire (SDQ) category, or diagnosis or treatment of a mental health disorder.

Anthropometric outcomes:

- Weight, kg;
- Height, cm;
- Head circumference, cm.

Cardiovascular outcomes:

- Systolic blood pressure, mm Hg;
- Diastolic blood pressure, mm Hg.

Metabolic outcomes (lipid profile):

- Cholesterol, mmol/L;
- High-density lipoprotein (HDL), mmol/L;
- Low-density lipoprotein (LDL), mmol/L;
- Triglycerides, mmol/L.

Respiratory outcomes:

- Asthma—history of asthma using questionnaires or asthma diagnosed by doctor in lifetime;
- Wheezing—wheezing in last 12 months or episodes of wheezing.

For the analysis, we selected studies presenting the cumulative risk of a given event occurring during the observation period, as well as studies presenting the point prevalence at a specific age. We excluded studies that used an ACS-initiating dose in both groups. In order to avoid duplicating data from the same populations, we excluded publications from the same databases in overlapping periods with same outcomes. In the case of studies where several follow-ups were available, we selected those with the longest follow-up period, but from the remaining ones, we selected non-overlapping outcomes. In the case of data divided according to the first ACS application in relation to gestational age, the data were summarized for analysis. In cases where the study included three groups—premature infants with and without prior ACS therapy and full-term infants without ACS therapy—premature infants without ACS therapy were selected as the control group. In the case of data available for cohorts of healthy women and women suffering from hypertension, for example, the cohort of healthy women was selected for analysis.

Continuous data were converted to a uniform unit and were converted into mean and standard deviation [mean (SD)]:

- If the data was presented as a median with quartiles [median (Q1, Q3)], the value was converted according to method presented by Luo et al. [42] and Wan et al. [43] using an available calculator without checking the skewness.
- If the data was presented as a mean (SE), the value was converted using the formula: $SD=\sqrt{N}\times SE$.

If case numbers were reported as percentages, they were converted to absolute values using standard rounding rules.

### 4.3. Quality Assessment

The quality of randomized clinical trials was evaluated using the Cochrane Collaboration’s tool [44], assessing random sequence generation, allocation concealment, blinding of participants and personnel, blinding of outcome assessment, incomplete outcome data, selective reporting and other bias, each rated as low, high or unclear risk. The quality of cohort studies was assessed using the Newcastle–Ottawa Scale (NOS) [45].

### 4.4. Statistical Analysis

Statistical analysis of the data was performed in R (version 4.2.2). To compare the effect of ACS exposure compared to non-exposure, the relative risk (RR) with a 95% confidence interval (CI) was calculated for dichotomous outcomes and the mean difference (MD) with 95% CI was calculated for continuous outcomes. A random effects model was used to calculate effect sizes. In subgroup analyses, the “childhood” subgroup included studies of children up to 10 years of age, while the “adolescence and adulthood” subgroup included studies of people aged 14 years and older and the “adulthood” subgroup included studies of people aged 23 years and older. I^2^ statistic was used to evaluate the heterogeneity of studies: I^2^ < 40% may not be important; 30% < I^2^ < 60% means moderate heterogeneity; 50% < I^2^ < 90% means substantial heterogeneity; I^2^ > 75% means considerable heterogeneity [46]. Publication bias was assessed using funnel plots, Peters’ regression test for dichotomous outcomes, and Egger’s regression test for continuous outcomes. The results were considered statistically significant at *p* < 0.05.

## 5. Conclusions

Although this meta-analysis suggests an increased risk of certain long-term neurological complications following exposure to prenatal corticosteroids, this evidence comes primarily from observational studies and may contain residual confounding factors. Prenatal corticosteroids remain a cornerstone of obstetric care due to their well-documented short-term benefits for newborns; however, the observed long-term associations warrant cautious interpretation. These findings underscore the need for a careful assessment of the benefit–risk ratio in clinical practice and highlight the importance of further, well-designed studies with long-term follow-up, standardized definitions of exposure, and better control of confounding factors to better elucidate potential long-term effects on neurological outcomes.

## Figures and Tables

**Table 1:** Characteristics of included studies.

Studies	Study Design	Country/City	Maternal Age (Years) Mean ± SD	Interventions	Participants	Follow-Up	Outcomes
Aviram et al., 2022 [6]	a population-based retrospective cohort study	Canada, Ontario	I: 30.2 ± 6.1 C: 30.2 ± 5.7	Information about the preparation, dose and timing of exposure is not recorded in the database, but according to Canadian guidelines, administration of betamethasone (2 × 12 mg intramuscularly, 24 h apart) or dexamethasone (4 × 6 mg intramuscularly, 12 h apart) is recommended between 24 and 34 weeks of gestation.	Late preterm singleton infants (34^0/7^–36^6/7^ gestational weeks)	5 years of age	suspected neurocognitive disorder, hearing impairment, visual impairment
Carballo-Magdaleno et al., 2011 [7]	a prospective study	Mexico	NA	A complete single course of antenatal steroids (betamethasone or dexamethasone).	preterm infants	12–36 months of age	weight, height, SBP, DBP
Dalziel et al., 2006 [8]	a follow-up of the Auckland Steroid Trial—double-blind, randomized, placebo-controlled trial	New Zealand, Auckland	NA	Women were randomized to receive an intramuscular injection of 6 mg betamethasone phosphate and 6 mg betamethasone acetate, or a placebo. The allocated treatment was repeated 24 h later if delivery had not occurred. After the first 717 women had enrolled, the dose of betamethasone in the intervention group was doubled.	all surviving term and preterm offspring	30 years	wheezing
Darlow et al., 2022 [9]	a population-based cohort follow-up study	New Zealand	NA	Mothers had received all or part of a course of ACSs.	infants with very low birth weight (<1500 g)	26–30 years of age	visual impairment, weight, height, SBP, DBP
Eriksson et al., 2012 [10]	a cohort study	Sweden	NA	Exposure to ACSs was estimated at the hospital level. Thus, if an infant was born at a maternity ward where routine ACS prophylaxis was offered, the infant was classified as exposed.	singleton infants born after gestational week 34^+0^	until 11 years of age or to date of death	cerebral palsy
Gyamfi-Bannerman et al., 2024 [11]	a prospective, follow-up study of a multicenter, double-blind, placebo-controlled trial conducted at MFMU Network centers of the National Institute of Child Health and Human Development	the United States	I: 29 ± 6.3 C: 29 ± 6.1	Consenting participants were randomly assigned to receive 12 mg of intramuscular betamethasone. A second, final dose was given if the participant had not delivered at 24 h.	term and preterm infants	6 years of age or older	autism spectrum disorders
Ho et al., 2025 [12]	a population-based retrospective cohort study	Taiwan	NA	ACS treatment is performed according to the American guidelines, either two 12 mg doses of betamethasone given parenterally 24 h apart or four 6 mg doses of dexamethasone parenterally every 12 h in pregnant women who are at risk of preterm labor. The timing of the first ACS administration was categorized according to GA: 22–27 weeks, 28–33 weeks, and 34–36 weeks.	singleton infants born at term (≧37^0/7^ weeks of gestation)	childhood	neurodevelopmental impairment, autism spectrum disorders, any mental or behavioral disorder
Kelly et al., 2012 [13]	a nested case–control study	the United Kingdom	NA	ACS use varied significantly between recruitment centers, consistent with local practice in the 1980s.	infants born preterm (birth weight <1850 g)	25 years (23 to 28 years of age)	SBP, DBP, cholesterol, HDL, LDL, triglycerides
LeFlore et al., 2002 [14]	a prospectively collected, previously validated computerized database and hospital and clinic records	the United States, Texas, Dallas	NA	Antenatal dexamethasone was given at the discretion of the attending obstetrician using guidelines. The antenatal dexamethasone dosing schedule was four 5 mg doses given intramuscularly every 12 h.	liveborn neonates with birth weight ≤1000 g	18 to 22 months of corrected age	neurodevelopmental impairment
Liu et al., 2012 [15]	the retrospective cohort study	China, Beijing	I: 26.9 ± 2.1 C: 27.7 ± 2.3	Antenatal dexamethasone 10 mg per day for 1 day or antenatal dexamethasone 10 mg per day for 2 consecutive days.	infants	6 years of age	weight, height, head circumference
McEvoy et al., 2017 [16]	follow-up of a randomized, double-blinded trial	the United States, Florida, Pensacola, and Oregon, Portland	NA	“Rescue AS” group received a course of ACSs (two 12 mg intramuscular injections of betamethasone 24 h apart); the “placebo” group received 2 doses of placebo.	preterm infants	1–2 years of corrected age	weight, height, head circumference, asthma, wheezing
Melamed et al., 2019 [17]	a retrospective cohort study	Canada, Ontario	I: 29.50 ± 5.87 C: 29.97 ± 5.48	Although information about the preparation, dose and timing of exposure are not recorded in the database, according to Canadian guidelines, administration of betamethasone (2 × 12 mg intramuscularly, 24 h apart) or dexamethasone (4 × 6 mg intramuscularly, 12 h apart) is recommended between 24 and 34 weeks of gestation.	singleton infants born at term (≥37^0/7^ weeks gestation)	5 years	suspected neurocognitive disorder, hearing impairment, visual impairment
Nixon et al., 2013 [18]	NA	the United States, North Carolina, Winston-Salem	NA	Antenatal corticosteroid therapy	singleton infants born prematurely with very low birth weight (<1500 g)	14 years of age	asthma, wheezing
Norberg et al., 2013 [19]	a cohort study	Sweden, Stockholm	I: 32.0 ± 5.1 C: 30.9 ± 5.3	The standard ACS treatment at the time and at this hospital consisted of an initial course of betamethasone 24 mg intramuscularly (8 mg q 8 h), followed by weekly courses of 12 mg betamethasone until delivery, or until pregnancy reached 34 gestational weeks.	infants	14 to 26 years	weight, height, SBP, DBP, cholesterol, HDL, LDL, triglycerides
Räikkönen et al., 2020 [20]	a population-based retrospective cohort study	Finland	I: 30.6 ± 5.8 C: 30.3 ± 5.3	Register does not include information on the number or timing of treatments. The treatment recommended by Finnish national guidelines consisted of betamethasone (12 mg) administered twice, 24 h apart throughout the study period. Repeated treatments were not recommended before 2009; after 2009, 1 repeat course could be considered when the risk of respiratory distress was high.	singleton preterm and term infants	median 5.8 (IQR, 3.1–8.7) years	any mental or behavioral disorder
Räikkönen et al., 2022 [21]	a population-based cohort study	Finland	Term I: 30.1 ± 5.8 C: 30.3 ± 5.4 Preterm I: 31.0 ± 5.7 C: 30.4 ± 5.7	Data were not available on the number of treatments or their timing. The Finnish national guidelines recommended betamethasone, 12 mg, administered twice, 24 h apart throughout the study period. Repeated treatments were not recommended before 2009; after 2009, 1 repeated course could be considered when the risk of respiratory distress was high.	singleton preterm and term infants	median of 5.8 (IQR, 3.1–8.7) years	cerebral palsy, autism spectrum disorders
Sultana et al., 2025 [22]	a pilot follow-up cohort study of participants from the double-blind, individually randomized WHO ACTION-I trial in Sylhet, Bangladesh	Bangladesh, Sylhet	I: 23.9 ± 5.3 C: 23.5 ± 5.3	Women were randomized to a regimen of intramuscular injections of 6 mg dexamethasone or placebo administered every 12 h, to a maximum of four doses (one or two courses).	infants who had survived to 28 completed days after birth	5 years (ages between 4.75 and 5.5 years of corrected age)	neurodevelopmental impairment, any mental or behavioral disorder, weight, height, head circumference
Ushida et al., 2020 [23]	a population-based retrospective cohort study	Japan	I: 31.4 ± 5.3 C: 31.0 ± 5.5	According to Japanese obstetrical guidelines, administration of ACSs (injection of 12 mg of betamethasone intramuscularly followed by a repeat injection 24 h later) is recommended. Although information on the type of ACS, dose of ACS, and administration-to-birth interval in each case was not included in the database, the majority of women in this study would have received a single course of betamethasone. Patients who received only one ACS injection until parturition were allocated to the ACS group.	preterm neonates born weighing ≤1500 g at 24–31 weeks of gestation admitted to the neonatal intensive care units (NICUs)	3 years of age	hearing impairment, visual impairment, cerebral palsy, neurodevelopmental impairment
de Vries et al., 2008 [24]	a retrospective matched-cohort study	the Netherlands, Utrecht, Leiden, Amsterdam and Zwolle	NA	2 × 12 mg intramuscular injections of betamethasone with an interval of 24 h between injections.	preterm infants born at less than 32 weeks of gestation	7 to 10 years of age	SBP, DBP
Walters et al., 2024 [25]	a follow-up of the Auckland Steroid Trial— double-blind, randomized, placebo-controlled trial	New Zealand, Auckland	NA	Women were randomized to receive either betamethasone 12 mg intramuscularly (6 mg of short-acting betamethasone phosphate and 6 mg long-acting betamethasone acetate), repeated at 24 h, or an identical-appearing control (standard treatment dose). The control treatment contained 6 mg cortisone acetate which has glucocorticoid potency of one-seventieth of the active treatment and an identical appearance. The dose of the betamethasone and control treatments were both doubled (twice standard treatment dose) in October 1972.	all surviving term and preterm offspring	50 years	weight, height, asthma
Walters et al., 2024 [26]	a follow-up of the Auckland Steroid Trial— double-blind, randomized, placebo-controlled trial	New Zealand, Auckland	NA	6 mg betamethasone sodium phosphate and 6 mg betamethasone acetate by intramuscular injection repeated after 24 h. In October 1972, the dose of the betamethasone and control treatments was doubled for the remainder of the trial.	all surviving term and preterm offspring	50 years	any mental or behavioral disorder
Wong et al., 2014 [27]	a retrospective population-based study	New South Wales and the Australian Capital Territory	Complete course I: 29.6 ± 6.7 C: 27.6 ± 6.7	ACS regimens consist of betamethasone, 2 × 12 mg given intramuscularly 24 h apart, or dexamethasone, 4 × 6 mg given intramuscularly 12 h apart. A complete course of antenatal steroids was defined as receipt of all doses 48 h to less than 7 days before delivery; infants who received steroids more than 7 days before birth were also classified as having a complete course. An incomplete course was defined as steroid exposure of less than 24 h before delivery.	infants admitted to NICUs born at less than 29 weeks gestational age	2–3 years of age	hearing impairment, visual impairment, cerebral palsy, neurodevelopmental impairment, weight, height, head circumference

NA—not applicable; ACSs—antenatal corticosteroids; GA—gestation age; SBP—systolic blood pressure; DBP—diastolic blood pressure; HDL—high-density lipoprotein; LDL—low-density lipoprotein.

## Data Availability

All data generated or analyzed during this study are included in this published article and its Supplementary Information Files.

## Supplementary Materials

The following supporting information can be downloaded at https://www.mdpi.com/article/10.3390/ph19071098/s1, Figure S1. Quality assessment of included RCTs; Table S1. Quality assessment of cohort studies based on Newcastle-Ottawa scale; Figure S2. Funnel plots for the association between ASC exposition and outcomes. The PRISMA checklist.

## Abbreviations

The following abbreviations are used in this manuscript:

ACS	antenatal corticosteroid
WHO	World Health Organization
RCT	randomized controlled trial
HDL	high-density lipoprotein
LDL	low-density lipoprotein
RR	relative risk
CI	95% confidence interval
MD	mean difference
SD	standard deviation
