# Supplementary material for: Antenatal Corticosteroids: Short-Term Gains, Long-Term Questions—A Meta-Analysis
Source: Pharmaceuticals (Basel). 2026 Jul 17;19(7):1098. doi: 10.3390/ph19071098 (PMC13414703; doi:10.3390/ph19071098)
Supplement: Supplementary file 1 [file pharmaceuticals-19-01098-s001.zip › pharmaceuticals-4417303-supplementary.pdf]

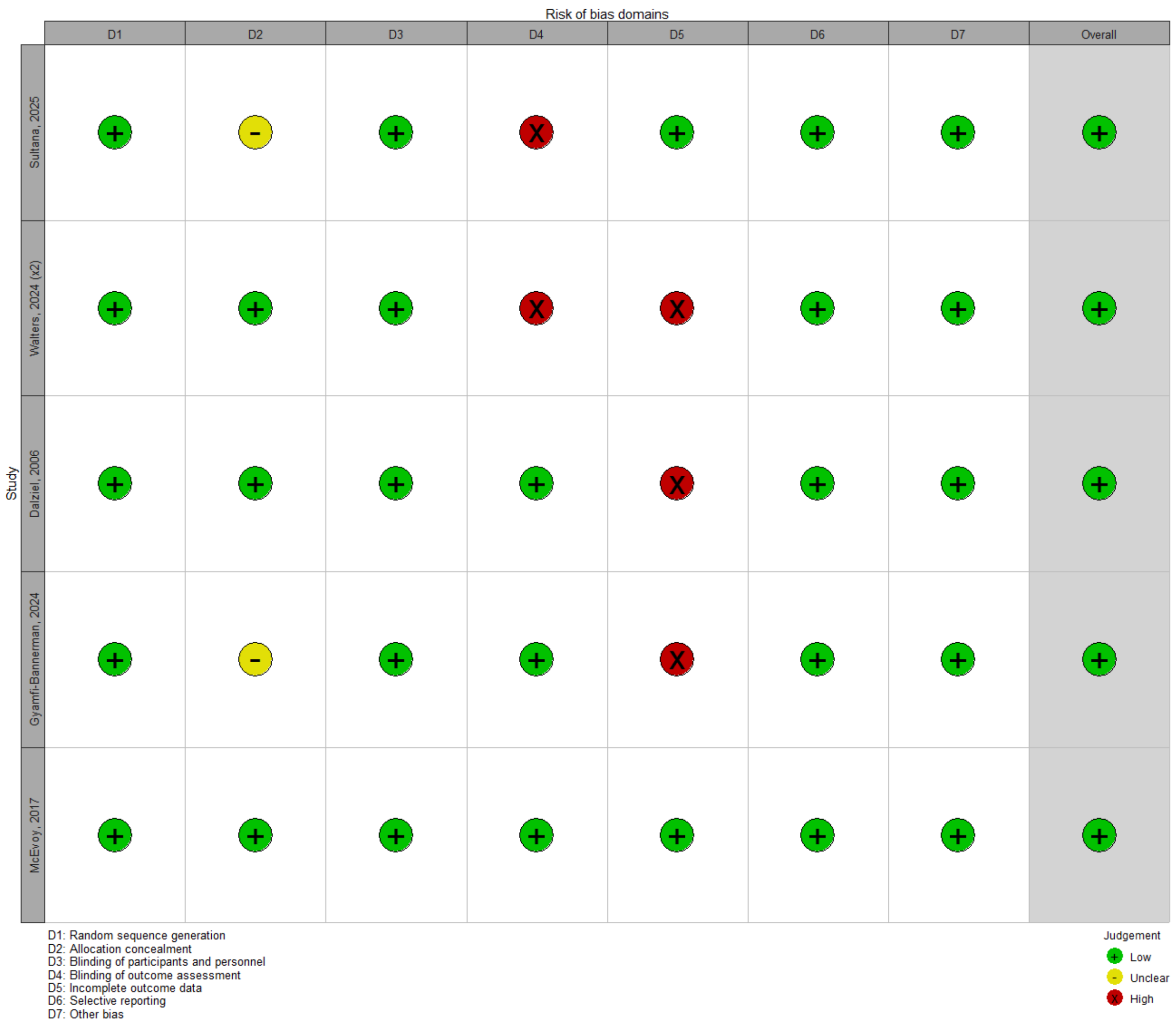

Figure S1. Quality assessment of included RCTs [8, 11, 16, 22, 25, 26].

Table S1. Quality assessment of cohort studies based on Newcastle-Ottawa scale.

	Selection					Outcome			
**Studies**	**Representativeness of the exposed cohort**	**Selection of the non-exposed cohort**	**Ascertainment of exposure**	**Demonstration that outcome of interest was not present at start of study**	**Comparability**	**Assessment of outcome**	**Was follow-up long enough for outcomes to occur**	**Adequacy of follow-up of cohorts**	**Quality**
Aviram, 2022 [6]	★	★	★	★	★★	★	★	★	good
Ushida, 2020 [23]	★	★	★	★	★	★	★		good
Liu, 2012 [15]	★	★	★	★	★★		★	★	good
Kelly, 2012 [13]	★	★	★	★	★★	★	★		good
Wong, 2014 [27]	★	★	★	★	★	★	★	★	good
Nixon, 2013 [18]	★	★	★	★	★	★	★	★	good
LeFlore, 2002 [14]		★	★	★	★	★	★	★	good
Räikkönen, 2020 [20]; Räikkönen, 2022 [21]	★	★	★	★	★★	★	★	★	good
de Vries, 2008 [24]	★	★	★	★	★	★	★	★	good
Eriksson, 2012 [10]	★	★	★	★	★★	★	★	★	good
Darlow, 2022 [9]	★	★	★	★	★★	★	★	★	good
Melamed, 2019 [17]	★	★	★	★	★★	★	★	★	good
Carballo-Magdaleno, 2011 [7]		★	★	★	★	★	★	★	good
Norberg, 2013 [19]		★	★	★	★★	★	★	★	good
Ho, 2025 [12]	★	★	★	★	★★	★	★	★	good

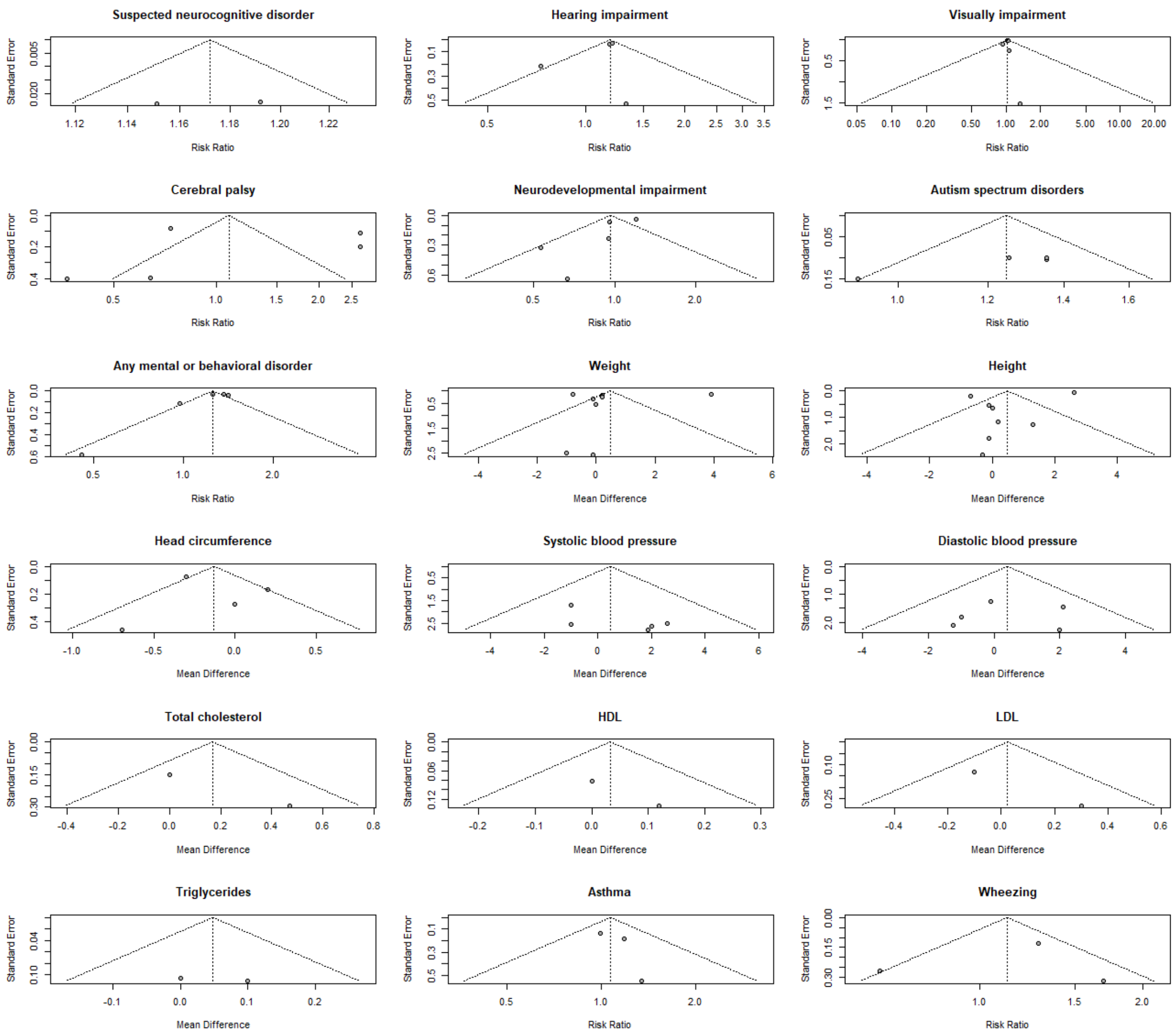

Figure S2. Funnel plots for the association between ASC exposition and outcomes.
